# Professionalising patient safety? Findings from a mixed-methods formative evaluation of the patient safety specialist role in the English National Health Service

**DOI:** 10.1177/13558196241268441

**Published:** 2024-08-02

**Authors:** Graham Martin, Robert Pralat, Justin Waring, Mohammad Farhad Peerally, Tara Lamont, on behalf of the Patient Safety Specialist Formative Evaluation Study Group

**Affiliations:** 1 Director of Research, THIS Institute, 2152 University of Cambridge, Cambridge, UK; 2 Research Associate, THIS Institute, 2152 University of Cambridge, Cambridge, UK; 3 Professor of Medical Sociology and Healthcare Organisation, Health Services Management Centre, 1724 University of Birmingham, Birmingham, UK; 4 Associate Professor, Social Science Applied to Healthcare Improvement Research Group, 4488 University of Leicester, Leicester, UK; 5 7423 Senior Advisor, THIS Institute, University of Cambridge, Cambridge, UK

**Keywords:** patient safety, health care improvement, National Health Service

## Abstract

**Objectives:** While safety-dedicated professional roles are common in other high-risk industries, in health care they have tended to have a relatively narrow, technical focus. We present initial findings from a mixed-methods evaluation of a novel, senior role with responsibility for leadership of safety in English National Health Service organisations: the patient safety specialist. **Methods:** We conducted interviews with those responsible for designing, developing and overseeing the introduction of the role. We also carried out a national survey of current patient safety specialists. Data collection and analysis focused on the rationale for the role, its theory of change, and experiences of putting the theory into practice. **Results:** Interview participants articulated a clear theory of change for the role, highlighting ways in which the focus of the role, the seniority, responsibility and influence of role holders, and the expertise they brought might result in better safety management and speedier implementation of initiatives to manage risk and improve safety. Survey respondents had mixed experiences of the role to date, particularly in terms of material and symbolic support from their organisations. Together, findings from the two datasets indicated the need for a careful balance between strategic and operational activities to secure impact for patient safety specialists while ensuring they were embedded in the realities of clinical work as done—a balance that not all role holders found easy to achieve. **Conclusions:** The vision for the patient safety specialist role is clear, and supported by a plausible account of how the work of role holders might result in the intended objectives. The degree to which specialists are supported and resourced to deliver on these ambitions, however, varies markedly across organisations.

## Introduction

Patient safety remains a challenge in health care organisations worldwide.^1^ The reasons for this are well-documented—including the inherent uncertainties and riskiness of diagnosis and treatment, the complexity of health care delivery, and the importance of social and cultural influences on safety—but how best to address these issues is less clear.^2^ Optimism about the potential of technical and managerial approaches to improving patient safety has given way to a more nuanced appreciation of the challenges of intervention,^3^ and increased recognition of the need to attend to the social and organisational systems in which safety practices operate.^4,5^

In England, major failings of health care quality and safety have resulted in sustained policy attention,^6^ and in 2019 the National Health Service’s central leadership and improvement body, NHS England, published the *Patient Safety Strategy*.^7^ Drawing explicitly on developments in safety science in health care and other industries, such as the idea of Safety II,^8^ it set out a vision for improving safety by improving systems and culture, and developing capability, skills and methods for insight and improvement. Among the innovations introduced in the *Strategy* was the role of ‘patient safety specialist’. Intended to be ‘captains of the team’^9^ and ‘key leaders within the safety system,’^7^ patient safety specialists were to co-ordinate action across their organisations and work to embed evidence-based, scientifically informed approaches such as ‘systems thinking, human factors and just culture principles.’^7^ All NHS organisations in England were asked to identify one or more specialists by late 2020, amounting to at least the equivalent of one full time in a senior position.^9^

Safety-dedicated roles are common in other industries,^10–12^ and subject to increasing professionalisation.^13^ Effectively realised, they can provide organisations with an informed understanding of safety issues, independent of operational line management and with influence at senior levels.^14,15^ In health care, however, roles relating to safety have tended to have a more specific, technical focus.^16–18^ The patient safety specialist role thus represents a promising innovation, representing what Hale et al. call safety *professionals* rather than mere safety *practitioners*,^13^ but one to date untested in health care. This paper reports findings from the initial stages of a formative evaluation of the specialist role in England. In the tradition of theory-based evaluation,^19^ we focus in particular on developing an understanding of the programme’s ‘theory of change’—the mechanisms by which its introduction is expected to give rise to intended outcomes—and the potential impediments to realising that theory in practice. Drawing on interviews with the team responsible for developing and overseeing implementation of the role and on a national survey of patient safety specialists, we seek to elucidate key features of the role and how they are expected to contribute to its success. We seek to use the early experiences of role-holder to illuminate wider influences on the role’s likely impact.

## Methods

We examined the development and implementation of the patient safety specialist role through a formative evaluation involving four stages. The first and second stages, reported in this paper, comprised, (i) a series of qualitative interviews with individuals in predominantly national-level roles involved in the development, roll-out and oversight of the role, and (ii) a cross-sectional survey of individuals occupying the roles. The third and fourth stages, not reported here, involved focus groups with specialists and case studies of the work of a purposive sample of specialists, along with meeting observations and stakeholder workshops. The whole study was supported by an expert collaborative group of stakeholders, including representatives of NHS England, individuals working as specialists, patient and public representatives (known as patient safety partners),^7^ and representatives of other relevant organisations. The group provided guidance and advice throughout the study—for example, informing interview topic guides, assisting with questionnaire design and sampling, providing critical feedback on emergent findings, and drawing on their networks to facilitate data collection.

The qualitative interviews in the first stage were intended to develop a stronger understanding of the theory of change behind the role, building on outlines in policy papers,^7,9,20–22^ and to collate reflections on learning from the implementation of the role. Potential participants were identified by the NHS England patient safety team, and through a process of ‘snowball’ sampling, whereby participants suggested others who might also offer relevant insights. Participants were provided with written information on the study and asked for consent for an audio-recorded semi-structured interview. Topic guides covered the thinking behind the role, expectations regarding how it would result in change, and experiences of implementation to date. Fifteen individuals were interviewed. Most interviews took place between September and November 2022 (with one additional interview in July 2023). They lasted 43-65 minutes (median 52 minutes) and were professionally transcribed. Analysis was based on the constant comparison approach.^23^ It involved initial line-by-line coding of the first three transcripts, resulting in the generation of a coding framework. All transcripts were then coded to this framework, and there followed successive refinement of initial codes into higher-level themes based on re-reading of data.

The survey sought to obtain information on the demographic and professional profiles of patient safety specialists, the ways the role had been implemented by individual NHS organisations, and the views of specialists on their experiences to date and hopes for the role. The questionnaire was informed by existing documentation and the wider academic literature, by analysis of interview data, and by the expert collaborative group. It was developed iteratively through discussion and testing within the research team and collaborative group. The questionnaire was not piloted, but the final version was subjected to extensive user testing before being administered through Thiscovery (https://www.thiscovery.org). It included a range of categorical, ordinal and open-response questions relating to participants’ backgrounds, experiences, day-to-day work and expectations for the role (see the online supplement). Participants were recruited through an email link circulated by NHS England to its distribution list of all patient safety specialists in England. Those following the link were provided with written information on the study and asked through initial screening questions to confirm their status as patient safety specialists and provide consent. No identifying data were collected. The survey was live from December 2022 to March 2023. The link to the survey was sent to 800 patient safety specialists; 194 (24%) completed the screening questions, of whom 184 (23%) proceeded to the questionnaire.

Qualitative data were coded inductively by common themes. Quantitative data were analysed in Excel using a range of descriptive and analytical statistical methods; in this paper, only descriptive statistics are reported.

## Results

We present our findings in three parts. First, we set out the key features of the role as articulated in interviews, and the mechanisms of their intended impact on patient safety. Next, we present data from the survey on the characteristics of patient safety specialists, including their backgrounds and how their roles had been implemented. Finally, we bring data from the interviews and survey together to examine the challenges and tensions involved in delivering the role, and the ways in which they might affect its impact.

Interview participants are referred to with the prefix ‘I’, while survey respondents are prefixed ‘R’.

### The role, its key features, and the anticipated effects

Interview participants cast the patient safety specialist role as crucial in ensuring system-wide improvements in patient safety. In particular, they emphasised the role’s importance in delivering goals of the national *Patient Safety Strategy*^7^—including its focus on improving systems and culture, and its commitment to exploiting advances in thinking in broader safety science and good practice in other industries. This included overseeing the delivery of specific initiatives, such as a new approach to investigating patient safety incidents (the Patient Safety Incident Response Framework, PSIRF), and the broader task of advancing the thinking encapsulated in concepts such as Safety II:

This is part of a new approach to safety, which is more proactive and changes and shifts, actually, the culture to a world where people are acting on safety issues. One, proactively, trying to prevent them, but secondly, working in real-time and not spending months doing highly bureaucratic processes, coming to a conclusion long after everyone’s forgotten about the whole thing and isn’t interested. (I3)

We also have an expectation that the specialists become, or are, our delivery vehicle for the *Strategy*, … employing all of those different ideas, concepts, and approaches, that I described around proactivity, and Safety II, and systems thinking, and human factors. (I1)

Central to this ambition was creating a role with senior oversight for patient safety within an organisation, similar to those found in some other sectors,^11,14^ with a purview exceeding individual, technical issues, and apprehending the interdependencies crucial to safety in a complex system:

Medication safety officers, it was medication issues. And the device safety officers, was devices, anything that is not medication. But then there are so many other things in patient safety, like culture, even the incident reporting, and all those other things that don’t fit into medication or device. So where does that sit and who has the responsibility for that? (I4)

This in turn implied both advanced understanding of patient safety concepts, such as systems thinking, and an ability to influence people and practices across an organisation. Therefore, both expertise and seniority formed key features of the specialist role:

The patient safety specialist, right from the start, was seen as a key part of the *Strategy*, because we’d picked up already from somewhere. We’d had a view from industry in the past about the importance of having that real leadership role in safety. … It’s that system focus—we’ve never had that before. (I5)

In bringing together these qualities and responsibilities in a focused, senior role, participants anticipated that specialists would be able to pursue several consequential activities for patient safety. First, they would be able to lead and co-ordinate existing safety efforts—both within organisations and across wider health economies:

Specialists need to be available at the system level as well, if they’re going to function effectively at the provider level, because so much of the safety issues and concerns we see happen at the point of transition or cross pathways between organisations. (I12)

At the same time, participants indicated that, in some instances, it might be acceptable or even desirable to employ more than one patient safety specialist—particularly in larger organisations. However, they emphasised the need to be explicit about the division of responsibilities among them and ensure this was clear to others within and outside the organisation.

Second, the presence of an identified, senior and expert individual in all NHS organisations would, it was suggested, permit more efficient and effective translation of patient safety interventions into routine practice. The national team would be able to rely on a standard single point of contact in disseminating both long-term strategy and urgent patient safety directives. Furthermore, specialists’ seniority and nuanced understanding of patient safety theory and practice would secure more reliable implementation, mitigate the risk of unintended consequences in complex systems, and close the feedback loop to the national team, providing it with insights around consistency of roll-out and the challenges faced in practice:

What we’ve seen in the past is we put an alert out saying, ‘There’s a problem with x, y and z,’ and it goes to somebody very junior, who doesn’t really know what to do with it, so they hand it to a few people who fix the bit. They swap out the old reagent for a new reagent and think their job is done and don’t understand the implication. … Patient safety specialists would understand that and would act appropriately in a way that hasn’t always been the case. (I3)

Thus, the theory of change behind the specialist role recognised the importance of both ‘top-down’ and ‘bottom-up’ communication to effective implementation, and ensuring that standards and directives were informed by a thorough understanding of the realities of ‘work as done’:

If you’re going to effect change, … you’ve got to open up the lines of communication. And rather than just pushing emails out from the centre, rather than just giving them stuff to do, I think the problem was that we weren’t hearing, always. … It’s allowed a dialogue with a greater number of experts in patient safety. (I9)

Third, lateral communication was identified as a core ingredient in the role. The mutual support and opportunities for knowledge-sharing afforded by a network of specialists would enhance their ability to address patient safety problems in their organisations, leavening the ‘know-what’ offered by top-down communication with the ‘know-how’ offered by peers:

It is really that sharing of information and the sharing of good practice and what’s worked well, whilst acknowledging [that] what works in one organisation isn’t necessarily going to translate to another. (I12)

Achieving these ambitions for the specialist role, participants noted, would require specific attributes of those occupying it. Participants were agnostic regarding professional affiliation: they suggested that relevant expertise might stem from a range of clinical and non-clinical backgrounds. Developing the skills and competencies of those in the roles was seen as important, and patient safety specialists would benefit from advanced training in various aspects of safety science, through the parallel introduction of the Patient Safety Syllabus, which was to complement basic training in safety for all NHS staff with higher-level training for patient safety specialists and others in key roles:

That’s the idea, that we would have this cohort of people—up to roughly 800, I think we’ve got at the moment—who are all trained to the equivalent of, or have actually received training in, level three and four of the Syllabus. And we therefore have a basis and a foundation level of knowledge for all of those people working in those roles across the system. (I1)

Besides expertise and experience in patient safety, and managerial competence in implementing complex projects, participants pointed towards the need for more than just effective delivery skills if changes in cultures and systems were to be achieved. Again echoing the language of Safety II, they highlighted for example the importance of moving beyond a focus on compliance and assurance, and of building relationships of influence across the organisation:

It still feels like people are feeling their way to a new way of delivery, and that the old school of, ‘Well, as long as I’ve audited it, and as long as I’ve kept the minutes of my safety committee, and as long as I’ve got a good panel and terms of reference up to date, then I’m in the clear.’ Moving away from that is hard, and then I think what it comes down to is just interpersonal relationships: who’s influencing you. (I11)

To some extent, participants reflected, this influence would arise from formal hierarchical position, and many pointed towards the need for specialists to be appointed at a senior grade, with direct access to organisations’ executive leadership teams. But, equally, they noted, influence was not reducible to seniority, and authority would also come from specialists’ personal style and willingness to engage in difficult conversations:

The idea is that this is a specialist who is an expert in the field of safety and who has the ability to influence and have the ear of the exec. … [But you also] need to be able to put some challenges in when people are doing things that are making operational sense but may not make patient safety sense, and to actually have your voice heard. (I6)

### The characteristics of patient safety specialists

Table 1 presents information on the demographic and professional characteristics of survey respondents. The majority (60%) came from non-medical clinical backgrounds, notably nursing and midwifery. Most were appointed at senior grades (only three per cent of those appointed were below Band 8a, the typical minimum point at which middle-management roles in the NHS are graded). The gender and ethnic profile of participants (75% female; 90% white) broadly reflected the characteristics of this tranche of the NHS workforce.^24,25^

**Table 1.** Personal and professional characteristics of survey participants (*N* = 184).

	*n*	%^a^
Sex		
Female	119	75
Male	39	25
Prefer not to say/no response	26	
Ethnicity		
Asian or Asian British	3	2
Black, Black British, Caribbean or African	3	2
Mixed or multiple ethnic groups	9	6
White	144	90
Other ethnic group	1	1
Prefer not to say/no response	24	
Occupational background^b^		
Allied health professionals, healthcare scientists, scientific and technical	24	
Medical and dental	14	
Ambulance (operational)	7	
Commissioning	12	
Registered nurses and midwives	89	
Nursing or healthcare assistants	3	
General management	116	
Other	3	
Occupational background type		
Clinical, excluding medical and dental	110	60
Medical or dental	14	8
Non-clinical managerial background	55	30
Other	5	3
Organisation type		
NHS acute provider	67	36
NHS mental health provider	17	9
NHS community provider	11	6
NHS mental health and community health provider	12	7
NHS integrated provider of acute, community and/or mental healthcare	15	8
NHS ambulance service	8	4
NHS specialist provider	2	1
Integrated Care Board	36	20
Independent-sector provider (for profit)	6	3
Independent-sector provider (not for profit)	5	3
NHS England (including NHS England regional teams)	4	2
Other	1	0
Time in post		
Less than 3 months	14	9
At least 3 months but less than six	14	9
At least than 6 months but less than 1 year	22	14
At least 1 year but less than two	65	40
2 years or more	47	29
No response	22	
Pay band		
Agenda for Change (AfC)^c^ Grade 7	5	3
AfC Grade 8a	43	28
AfC Grade 8b	33	22
AfC Grade 8c	31	20
AfC Grade 8d	22	14
AfC Grade 9	4	3
Executive senior manager	2	1
Consultant-grade doctor	12	8
Prefer not to say/no response	32	

^a^Figure excludes those not responding or selecting ‘prefer not to say’.

^b^Percentages not given because participants could select more than one category.

^c^Agenda for Change (AfC) is the grading scheme for most non-medical staff in the NHS.

**Figure 1.**
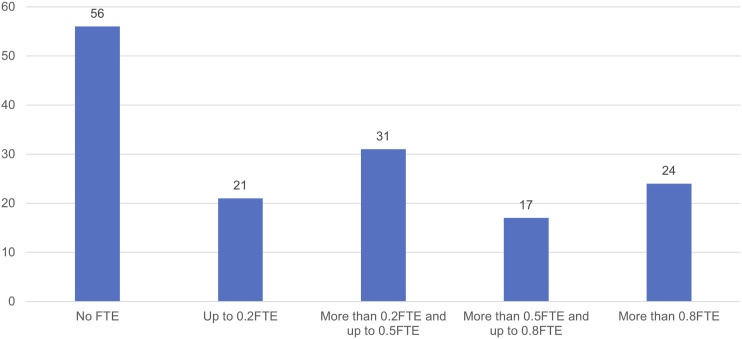
Proportion of full-time equivalent (FTE) working time formally allocated to patient safety specialist role (35 participants did not respond to this question).

**Figure 2.**
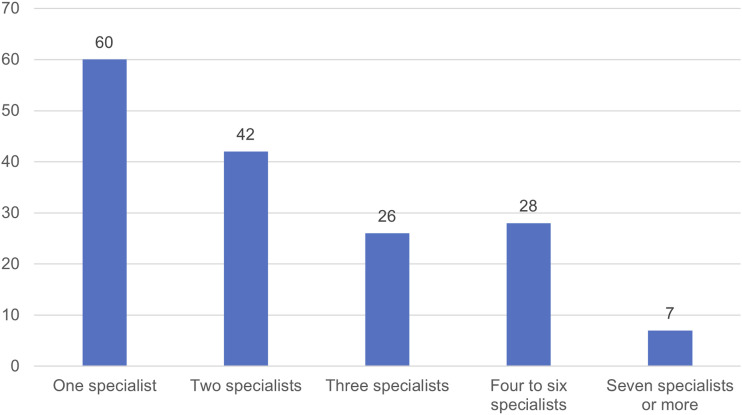
Number of patient safety specialists (including the participant) employed in the participant’s organisation (21 participants did not know or did not respond to this question).

Participants varied both in the amount of time they had to dedicate to the patient safety specialist role (Figure 1) and in the total number of specialists designated in their organisation (Figure 2). Only 24 (16%) of respondents reported that at least 80% of their time was formally allocated to delivering the role, with 56 (38%) reporting that no time was officially protected for the role—though some noted in free-text comment that, in practice, they were able to use more of their time. Most (103 or 63%) reported that they were not the sole specialist employed by their organisation; in acute hospital organisations, this rose to 79% (49/62). Across the sample, 35 (21%) stated that they were one of at least four specialists. This finding reflects interviewees’ suggestions that the responsibilities of the role might best be distributed among more than one person. However, 41 participants (22%) indicated both that they were the only patient safety specialist in their organisation and that they had at most 80% of their time allocated to the role. Of these 41, 20 had 20% of their time or less formally allocated. The ambition set out in policy that the role ‘should be full time, … focusing solely on patient safety’^9^ appears not to have been realised in all organisations.

Participants were asked to indicate their agreement with statements about their role and its likely impact (Table 2). Participants were moderately optimistic about the role’s potential (58% of those responding agreeing or strongly agreeing that they could make a positive difference to patient safety; statement 12). This varied by organisation type: respondents in integrated care boards (ICBs), which are responsible for commissioning and coordinating care in an area but not for care delivery, appeared to be rather more cautious than their counterparts in provider organisations, with 28% (10/36) disagreeing or strongly disagreeing that they could make a positive difference, compared to only 11% (10/94) of those in provider organisations. Most (72%) agreed or strongly agreed that they could access their organisations’ boards when they needed to (statement 5), though views were more mixed on how well they were supported in their role (42% agreeing or strongly agreeing they were well supported; 30% disagreeing or strongly disagreeing; statement 11). There was a notably negative skew to the statement regarding adequacy of time available for the role (51% disagreeing or strongly disagreeing that their organisation allocated sufficient time; statement 3), reflecting the limited allocation of time available to many participants (Figure 1).

**Table 2.** Number of responses to questions regarding the role, support available, and likely impact of patient safety specialists.

Statement		Strongly disagree	Disagree	Neutral	Agree	Strongly agree	No response
1	I am clear on the purpose of my role as a patient safety specialist	1	18	43	89	28	5
2	The objectives set by my organisation for the role of patient safety specialist are appropriate	13	30	75	41	20	5
3	My organisation allocates sufficient time to its patient safety specialist(s) to achieve the objectives of the role	44	47	45	33	10	5
4	My organisation’s board gives sufficient priority to patient safety	12	35	49	61	21	6
5	I Have access to a member of the board when I need it	11	12	26	54	75	6
6	In my organisation, most of the people who need to know are aware of my role as a patient safety specialist	14	34	57	53	21	5
7	Patient safety partners in my organisation have a clearly defined role in improving patient safety	50	38	55	26	6	9
8	My role as a patient safety specialist is very similar to the role I held immediately before	18	36	30	65	29	6
9	Most of the things I do as a patient safety specialist could only be done by someone with at least my level of expertise or experience	5	17	18	99	36	9
10	Most of the things I do in my role as a patient safety specialist will have a useful impact on patient safety	1	9	27	104	36	7
11	I feel well supported in my role as a patient safety specialist	20	34	50	52	22	6
12	I am confident I can make a positive difference to patient safety in my role as a patient safety specialist	6	23	45	75	29	6

### Challenges and tensions in delivering the role

The interviews and the free-text survey responses identified both organisational receptiveness and the capacity available to specialists as challenges for patient safety specialists. The impression of interviewees was that organisations had varied markedly in their responses to the prompt to appoint specialists, some having ‘got it’ (I5) while others had not. Survey participants confirmed the range of organisational approaches: ‘The seniority of my role … enables routine access to Board members’ (R3); ‘I do not have direct access to the Lead Exec and feel that my role as PSS is not really understood’ (R111). Reflecting their less optimistic views on their ability to make a positive difference, patient safety specialists in ICBs also noted particular challenges in putting the role into practice, given the distinctive function of their organisations: ‘Lack of national guidance about what the role should be in an ICB. All of the webinars and communications focus on providers’ (R122); ‘The role in commissioning organisations has never been defined. Therefore I’m really unclear as to what is expected of me’ (R11). Interview participants noted that organisational commitment was likely to have a bearing on role holders’ capacity:

I don’t know whether individual organisations have found funding for patient safety specialists or […] whether they would just put it on to somebody’s current job role. That to me isn’t a problem necessarily, as long as [they] have the time, the headspace, to be able to do the job. (I2)

Survey participants confirmed that in many cases, the work of patient safety specialist had indeed been ‘bolted on’ to their existing roles—sometimes without a commensurate reduction in their existing responsibilities. As noted above, a significant minority had no protected time for their specialist role (Figure 1), and many participants noted in free-text responses the struggles they faced in doing the job justice: ‘This role is additional to my full-time role and therefore I feel I am not able to give it the time and importance I would like’ (R106); ‘This is a voluntary role on top of our job. This means the work we do must be squeezed into any available time’ (R57). Some noted the absence of resources, guidance and training, or the difficulty of accommodating wide-ranging priorities in their work. Others highlighted that day-to-day operational pressures could quickly erode time theoretically reserved for patient safety-related activity: ‘Constantly pulled to plug gaps in other areas, diverted away from role’ (R65). As an interviewee put it, ‘clinical care is always going to trump everything’ (I13).

Even those with more time allocated faced tensions between what might be termed the role’s ‘operational’ and ‘strategic’ elements. Interview participants were clear that the specialist role needed to be primarily a strategic one, and highlighted the risks that might be posed by becoming embroiled in detail, even if that detail was critical to patient safety:

[Specialists should be] not just leaders of process, but leaders of people. … If they get bogged down in the minutiae of, ‘Oh well, I’ve got to investigate this never event,’ or, ‘I’ve got to implement this alert,’ then they’ll never give you that leadership. They’ll always just be feeding the beast. (I11)

**Figure 3.**
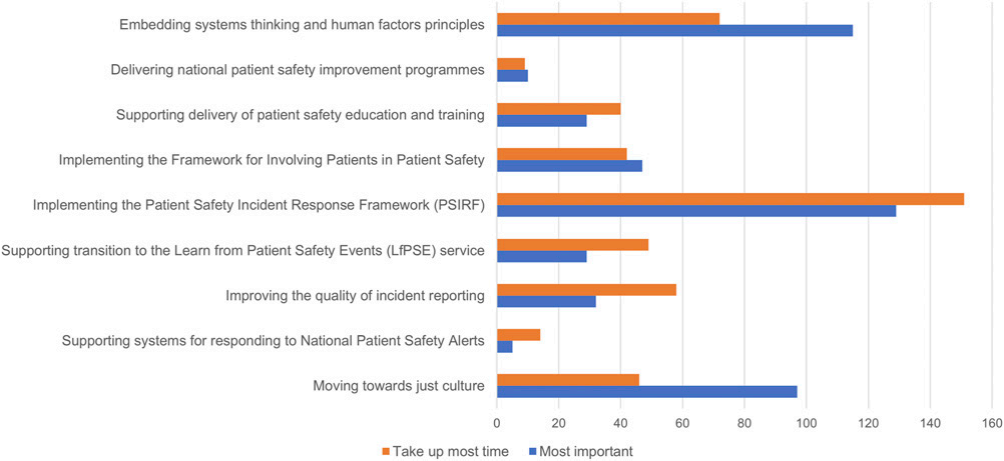
Priorities seen as among top-three most important (*N* = 179) and most time-consuming (*N* = 180), by number of survey participants.

Quantitative and qualitative survey data confirmed that achieving this balance was challenging. Survey participants were asked, in separate questions, to select from among nine priorities derived from national-level policy^7,20,22^ the three they saw as most important, and the three that consumed most time (Figure 3). While some (e.g. implementing PSIRF) score highly against both criteria, for others there was a notable divergence—especially in relation to priorities with a strong long-term or strategic dimension. ‘Embedding systems thinking and human factors principles’ was seen as a top-three priority by 64% (115/179) of participants but was among the top three most time-consuming for only 40% (72/180). Similarly, ‘Moving towards a just culture’ was among the top three most important for 54% (97/179) but in the top three most time-consuming for 26% (46/180). The reverse was true for ‘Improving the quality of incident reporting’, despite the cautions of interviewees. Many free-text comments confirmed the impact of this challenge on the value they saw in their role: challenges cited included ‘Exec[utive] focus on SIF [Serious Incident Framework] performance, counting incidents etc. rather than improvement’ (R20), and that ‘Too much of the role is focused on the incident that has already happened’ (R28).

Yet also apparent in the datasets was a sense that, although specialists should have clear strategic influence, presence and visibility, some involvement in operational detail was inevitable—even important. Besides the simple need to ensure that operationally focused but safety-critical work (such as implementing urgent alerts) was done effectively, participants also pointed towards the need for specialists to have a nuanced and realistic understanding of work as done, of the kind that could only arise from immersion in operational details:

I think it’s that everyday thing of, ‘We’ve got a particular issue, an event, an incident. We need to be on the floor.’ It’s the thing of, ‘Go to the floor and see what’s going on and see how you can support people in those situations,’ which is probably one of the more important things. (I3)

Survey respondents likewise highlighted the complementarity between presence at the ‘sharp end’ of care delivery and orchestrating strategy at the ‘blunt end’:

Working on the frontline adds credibility and also invaluable knowledge of how things are working and networking with multiple staff. However, the challenges are that project support is required to maintain timely progress and enable overarching leadership role, and this is minimal. (R3)

Where serious incidents occur on inpatient areas. then I often get asked to attend these wards to ensure the environment is operating safely and to provide assurance to senior and executive staff. This can, on occasions, lead to more in-depth pieces of improvement work being launched that can involve 6-8 weeks of focused work from myself. (R56)

Meaningful understanding of safety in practice and the ability to offer strategic oversight and influence thus sat in something of a creative tension, each informing the other.

Other tensions that were similarly difficult to reconcile were evident. While the introduction of the specialist role created a focal point for accountability for patient safety, participants emphasised the need to avoid overburdening an individual, and to ensure that responsibility for patient safety remained dispersed. Interviewees acknowledged that finding the right balance between these imperatives could be challenging:

The ask of patient safety specialists is quite big. … I think that’s the danger with not having a really clear idea in an organisation about who’s going to do the what, or who’s going to co-ordinate it, the doing. Because you can’t have the patient safety specialist overseeing all of the safety investigations, for instance, … [but] whoever is responsible for undertaking those needs to be able to take that intelligence through the patient safety specialist, in a way that informs the patient safety specialist's wider responsibilities. (I9)

Survey respondents described a range of ways in which they sought to address this challenge. Where organisations had appointed multiple specialists, labour was divided accordingly, sometimes along strategic-operational lines, sometimes by profession or directorate, and sometimes according to grade: ‘A nurse, governance lead and doctor sharing role is both a key positive and a challenge as role and time for each of us is ill-defined’ (R89); ‘I am a doctor and we also have a head of patient safety (nurse) who is a patient safety specialist. I think it is important to have representation from both. … I have 1 day a week (but do more!) for this role and it can be hard to balance the reactive part (support for investigations) with the more proactive part of safety’ (R60).

A final tension was between the need for regimented implementation of best practice and the need for flexibility, creativity and context-sensitivity in approach. Interviewees emphasised the importance of specialists’ work in implementing national directives. But they also noted the role of specialists in securing safety locally through an intimate knowledge of work as done and a consequent appreciation of the limits of top-down standardisation. Survey respondents, similarly, highlighted the importance of their work in implementing patient safety measures—and obtaining assurance that they were routine—and a less specified, more wide-ranging role in creating cultures in which safety could flourish:

I think we have changed the culture in our approach to learning from incidents and engaging directly with clinical teams – this is a good foundation for PSIRF but doesn’t actually get the implementation process done. (R70)

[The role] needs to be a dedicated high level patient safety ‘specialist’ – as per suggested person spec free to ‘roam with authority’ to act – my worry is that organisations don't ‘get’ the wider remit of the role. (R69)

## Discussion

Our findings indicate a clear vision for the patient safety specialist, a well-articulated idea about how specialists and their work might help to realise improvements in patient safety, and a range of challenges in realising the role. Reflecting learning from other industries,^10,11,18^ interview participants described how a senior role with expertise, accountability and oversight, supported by a network of peers, might combine to secure better co-ordination of safety efforts, more effective implementation of new initiatives, and more nuanced understanding of challenges in practice. Survey data indicated that organisations differed in their approach to implementing the role, with varying levels of resourcing. A majority of respondents felt they had insufficient time to deliver the objectives of the role, and many indicated that the support for and understanding of patient safety displayed by their organisation left much to be desired. Like other health care systems contending with ageing populations and post-pandemic operational backlogs,^26^ the English NHS faces severe resource pressures. However, reflecting findings from roll-out of other similar national patient safety initiatives,^2^ both the theory of change and the experiences of those in these new roles suggest that without dedicated time and organisational support for their work, patient safety specialists will struggle to achieve the impact envisaged.

Besides these practical challenges, both interviewees and survey respondents identified tensions in realising the role, many of which needed to be managed carefully. Strategic influence, for example, needed to be informed by a thorough understanding of operational reality, while patient safety expertise and responsibility needed to be diffused as well as concentrated. Similar tensions have been found in other sectors, where research has highlighted the need for example for safety professionals to be involved at the sharp end yet influential at the top of organisations,^15^ to retain an independence from operational matters that can sometimes be at odds with changing them,^14^ and to act as both specialists who bring technical expertise to safety management and generalists who can integrate multiple perspectives and competing priorities.^13^ Analogous roles in those other sectors, however, often operate as part of established safety management systems that are less common in health care organisations and which may offer safety professionals a framework both for activity and for organisational support.^27^ The challenges identified by participants in this study may reflect the recent introduction of the role, lack of capacity, or the absence (at least as yet) of a clear and consistent approach to managing safety in NHS organisations.

In some ways, these challenges perhaps reflect a broader tension in the work and responsibilities of patient safety specialists: between Safety I and Safety II. Safety professionals in other industries find themselves torn between safety management practices associated with Safety I, which offer a clear template and are often endorsed by organisational leaderships, and the principles of Safety II, which offer an attractive but less well codified, and potentially more challenging, alternative.^28^ Tensions between centralised control and dispersed accountability, between assurance and improvement, and between standardisation and flexibility were evident in the responses of interviewees and survey participants alike. To some extent they might reflect friction between the introduction of new ideas in policies such as the *Patient Safety Strategy*^7^ and prevailing organisational structures and processes that reproduce existing practices.^29^ But they also, perhaps, exemplify the challenges of a realisation of patient safety that makes the best of both approaches, and find practical ways of reconciling the two.^30,31^ Patient safety specialists find themselves in the daunting but, for some at least, exciting position of trying to take patient safety forward by incorporating new thinking without relinquishing the benefits of existing practices.

## Limitations

Our study has three main limitations. First, the survey response rate was low at 24%. While not an unusual figure for surveys of this kind, it does mean that inferences about the characteristics and views of the whole population of patient safety specialists should be made only cautiously. Relatedly, we did not ask survey respondents to indicate the organisation to which they belonged, and it is possible therefore that some organisations are over-represented while others are under-represented (which may have a particular impact on data relating to the numbers of patient safety specialists per organisation).

Second, the interviewee recruitment process may have resulted in an over-representation of those with more positive views. Since interviewees were recruited initially through NHS England, with subsequent snowball sampling, it may be that those more favourably disposed towards the initiative were selected, or that interviews content was influenced by participants’ awareness of NHS England’s role in assisting recruitment.

Third, both data-collection methods may be affected by social-desirability bias—that is, the tendency, unconscious or otherwise, to give responses that reflect what the interviewer, survey administrator or wider society expect to see (in this case, positivity about the potential of the new role).

## Conclusion

In examining the theory of change and realisation in practice of the novel patient safety specialist role, our study finds well-articulated ideas for how the role might bring about change but inconsistencies of implementation that may impede its effectiveness. Competing pressures on those in the role and mixed levels of organisational understanding and support suggest that key ingredients of the theory of change, such as its dedicated focus and senior-level influence, may be difficult to achieve. Wider tensions in delivery—for example, matching detailed understanding of the actual work involved with the role with a focus on strategy—reflect challenges found in similar roles in other industries. Learning from their adaptive work as they seek to manage these tensions will be critical in helping patient safety specialists make the best of the role.

## Supplemental Material


Supplemental Material - Professionalising patient safety? Findings from a mixed-methods formative evaluation of the patient safety specialist role in the English national health service
Supplemental Material for Professionalising patient safety? Findings from a mixed-methods formative evaluation of the patient safety specialist role in the English national health service by Graham Martin, Robert Pralat, Justin Waring, Mohammad Farhad Peerally and Tara Lamont in Journal of Health Services Research & Policy.
