# Supplementary material for: Professionalising patient safety? Findings from a mixed-methods formative evaluation of the patient safety specialist role in the English National Health Service
Source: J Health Serv Res Policy. 2024 Aug 2;30(1):40–51. doi: 10.1177/13558196241268441 (PMC11673301; doi:10.1177/13558196241268441)
Supplement: Supplemental Material - Professionalising patient safety? Findings from a mixed-methods formative evaluation of the patient safety specialist role in the English national health service [file sj-pdf-1-hsr-10.1177_13558196241268441.pdf]

Questionnaire used in national survey of patient safety specialists

Q1. In what type of organisation do you undertake your role as Patient Safety Specialist?

▼ NHS acute provider (providing largely hospital-based services)
NHS mental health provider
NHS community provider (providing services such as district nursing, health visiting)
NHS mental health and community health provider
NHS integrated provider including acute, community and/or mental healthcare
NHS ambulance service
NHS specialist provider (providing services such as specialist eyecare or cancer treatment)
Integrated Care Board
Independent-sector provider (for profit)
Independent-sector provider (not for profit)
NHS England (including NHS England regional teams)
Other (please state)

Display Q1a if:
If Q1 = Other

Q1a. If you answered ‘Other’, how would you describe your organisation?

____

Q2. What is your occupational group? Tick all answers that apply.

- ☐ **Medical and Dental**
- ☐ **Ambulance (operational) (e.g. paramedic, ambulance technician)**
- ☐ **Registered Nurses**
- ☐ **Registered Midwives**
- ☐ **Nursing or Healthcare Assistants**

Allied Health Professionals / Healthcare Scientists / Scientific and Technical

- ☐ Occupational Therapy
- ☐ Physiotherapy
- ☐ Radiography
- ☐ Pharmacy
- ☐ Clinical Psychology
- ☐ Other qualified Allied Health Professionals (e.g. dietetics, speech and language therapy)
- ☐ Support to Allied Health Professionals (e.g. support worker, therapy worker, therapy assistant)
- ☐ Other qualified Scientific and Technical or Healthcare Scientists (e.g. haematology, clinical biochemistry, microbiology)
- ☐ Support to Healthcare Scientists (e.g. technicians, assistants)

Management

- ☐ Commissioning
- ☐ Patient safety, governance, risk, quality assurance
- ☐ Other Management
- ☐ **Other – please describe:** ____

Q3. In the following question, we will ask you to **select or add** priorities which you see as **most important** to your role as Patient Safety Specialist, up to a maximum of **three.** To answer:

Please read the list of priorities below.

Add any you feel are missing.

Select the **three** which are the **most important** to your role as Patient Safety Specialist.

- ☐ Moving towards a just culture in your organisation, system or region
- ☐ Ensuring that systems thinking and human factors principles are embedded in all safety processes in your organisation, system or region
- ☐ Supporting local systems for planning and coordinating the actions required by National Patient Safety Alerts
- ☐ Improving the quality of incident reporting in your organisation, system or region
- ☐ Supporting your organisation's transition to the Learning from Patient Safety Events (LfPSE) service
- ☐ Implementation of the new Patient Safety Incident Response Framework (PSIRF) in your organisation, system or region
- ☐ Implementation of the Framework for Involving Patients in Patient Safety in your organisation, system or region
- ☐ Supporting the delivery of patient safety education and training in your organisation, system or region
- ☐ Delivery of national patient safety improvement programmes
- ☐ Other priority 1: ______________________________
- ☐ Other priority 2: ______________________________
- ☐ Other priority 3: ______________________________

Q4. Of the following priorities, which **three** do you **spend the most time on** in your role as Patient Safety Specialist? Again, to answer:

Please read the list of priorities below.

Add any you feel are missing.

Select the **three** which **you spend the most time on**.

- ☐ Moving towards a just culture in your organisation, system or region
- ☐ Ensuring that systems thinking and human factors principles are embedded in all safety processes in your organisation, system or region
- ☐ Supporting local systems for planning and coordinating the actions required by National Patient Safety Alerts
- ☐ Improving the quality of incident reporting in your organisation, system or region
- ☐ Supporting your organisation's transition to the Learning from Patient Safety Events (LfPSE) service
- ☐ Implementation of the new Patient Safety Incident Response Framework (PSIRF) in your organisation, system or region
- ☐ Implementation of the Framework for Involving Patients in Patient Safety in your organisation, system or region
- ☐ Supporting the delivery of patient safety education and training in your organisation, system or region
- ☐ Delivery of national patient safety improvement programmes
- ☐ Other priority 1: ____
- ☐ Other priority 2: ____
- ☐ Other priority 3: ____

Please indicate the extent to which you agree or disagree with each of the following 12 statements. **(1 = Strongly disagree, 5 = Strongly agree)**

Q5. I am clear on the purpose of my role as Patient Safety Specialist.

- ◯ 1: Strongly disagree
- ◯ 2: Disagree
- ◯ 3: Neither agree nor disagree
- ◯ 4: Agree
- ◯ 5: Strongly agree

Q6. The objectives set by my organisation for the role of Patient Safety Specialist are appropriate.

- ◯ 1: Strongly disagree
- ◯ 2: Disagree
- ◯ 3: Neither agree nor disagree
- ◯ 4: Agree
- ◯ 5: Strongly agree

Q7. My organisation allocates sufficient time to its Patient Safety Specialist(s) to achieve the objectives of the role.

- ◯ 1: Strongly disagree
- ◯ 2: Disagree
- ◯ 3: Neither agree nor disagree
- ◯ 4: Agree
- ◯ 5: Strongly agree

Q8. My organisation's board gives sufficient priority to patient safety.

- ◯ 1: Strongly disagree
- ◯ 2: Disagree
- ◯ 3: Neither agree nor disagree
- ◯ 4: Agree
- ◯ 5: Strongly agree

Q9. I have access to a member of the board when I need it.

- ◯ 1: Strongly disagree
- ◯ 2: Disagree
- ◯ 3: Neither agree nor disagree
- ◯ 4: Agree
- ◯ 5: Strongly agree

Q10. In my organisation, most of the people who need to know are aware of my role as Patient Safety Specialist.

- ◯ 1: Strongly disagree
- ◯ 2: Disagree
- ◯ 3: Neither agree nor disagree
- ◯ 4: Agree
- ◯ 5: Strongly agree

Q11. Patient Safety Partners in my organisation have a clearly defined role in improving patient safety.

- ◯ 1: Strongly disagree
- ◯ 2: Disagree
- ◯ 3: Neither agree nor disagree
- ◯ 4: Agree
- ◯ 5: Strongly agree

Q12. My role as Patient Safety Specialist is very similar to the role I held immediately before.

- ◯ 1: Strongly disagree
- ◯ 2: Disagree
- ◯ 3: Neither agree nor disagree
- ◯ 4: Agree
- ◯ 5: Strongly agree

Q13. Most of the things I do as Patient Safety Specialist could only be done by someone with at least my level of expertise or experience.

- ◯ 1: Strongly disagree
- ◯ 2: Disagree
- ◯ 3: Neither agree nor disagree
- ◯ 4: Agree
- ◯ 5: Strongly agree

Q14. Most of the things I do in my role as Patient Safety Specialist will have a useful impact on patient safety.

- ◯ 1: Strongly disagree
- ◯ 2: Disagree
- ◯ 3: Neither agree nor disagree
- ◯ 4: Agree
- ◯ 5: Strongly agree

Q15. I feel well supported in my role as Patient Safety Specialist.

- ◯ 1: Strongly disagree
- ◯ 2: Disagree
- ◯ 3: Neither agree nor disagree
- ◯ 4: Agree
- ◯ 5: Strongly agree

Q16. I am confident I can make a positive difference to patient safety in my role as Patient Safety Specialist.

- ◯ 1: Strongly disagree
- ◯ 2: Disagree
- ◯ 3: Neither agree nor disagree
- ◯ 4: Agree
- ◯ 5: Strongly agree

We'd like to understand your experience as a Patient Safety Specialist. The next two questions will help us to know what works well and what could be better.

Q17. What is your **biggest achievement** in your role as Patient Safety Specialist to date?

____

____

____

Q18. What are the **main challenges** you face in carrying out your role as Patient Safety Specialist effectively?

We'd like to find out a little more about who you work with and where your role fits in. The next few questions are about relationships with your colleagues.

Q19. What are the job titles of the **three people** (not including other Patient Safety Specialists) with whom you work most closely in your role as Patient Safety Specialist **within your organisation**?

- ○ 1 __________
- ○ 2 __________
- ○ 3 __________

Q20. What are the job titles of the **three people** (not including other Patient Safety Specialists) with whom you work most closely in your role as Patient Safety Specialist **outside your organisation**?

- ◯ 1 ________________________________
- ◯ 2 ________________________________
- ◯ 3 ________________________________

Q21. Are you the only Patient Safety Specialist in your organisation?

- ◯ Yes
- ◯ No
- ◯ Don't know

Display Q22 if:

Q21 = No

Q22. How many other Patient Safety Specialists (besides yourself) are employed in your organisation?

- ◯ 1 other Patient Safety Specialist
- ◯ 2 other Patient Safety Specialists
- ◯ 3 to 5 other Patient Safety Specialists
- ◯ 6 or more other Patient Safety Specialists
- ◯ Don't know

Q23. What is the job title of your line manager, or the person to whom you are immediately accountable in your role as Patient Safety Specialist?

____

Almost there! Just a few more questions about your job. This is so we have a good understanding of the Patient Safety Specialist role and the people who have been appointed to it.

Q24. How long have you been working in your current role as Patient Safety Specialist?

- ○ Less than 3 months
- ○ 3 – 6 months
- ○ 6 months – 1 year
- ○ 1 year – 2 years
- ○ 2 years or more

Q25. At what grade is your Patient Safety Specialist post banded?

- ◯ Agenda for Change (AfC) Grade 6 or lower
- ◯ AfC Grade 7
- ◯ AfC Grade 8a
- ◯ AfC Grade 8b
- ◯ AfC Grade 8c
- ◯ AfC Grade 8d
- ◯ AfC Grade 9
- ◯ Executive Senior Manager (ESM) (also known as Very Senior Manager or VSM)
- ◯ Staff-grade / associate specialist / specialty (SAS) doctor
- ◯ Training-grade doctor (e.g. specialist registrar)
- ◯ Consultant-grade doctor
- ◯ Other (please describe)

____

- ◯ Prefer not to say

Q26. Is the grade of your role as Patient Safety Specialist the same as the grade of the role you had immediately before you became Patient Safety Specialist?

- ◯ My previous role was at a lower grade
- ◯ My previous role was at the same grade
- ◯ My previous role was at a higher grade
- ◯ Other – please describe: ____
- ◯ Prefer not to say

Q27. How much of your time is **formally** allocated to your responsibilities as Patient Safety Specialist? Please enter as days per week or full-time equivalent, e.g. ‘two days a week’ or ‘40% FTE’.

____

Q28. What was the job title of the role you had immediately before becoming a Patient Safety Specialist?

____

Q29. Do you still hold that role alongside your Patient Safety Specialist role?

- ◯ Yes – all of it
- ◯ Yes – some of it
- ◯ No

Finally, we'd like to ask a few questions about you. This is so we know more about who Patient Safety Specialists are and how that relates to their experiences.

Display Q30 if:

Q1 ≠ NHS England (including NHS England regional teams)

Q30. In which region is your organisation located?

- ◯ East of England
- ◯ London
- ◯ Midlands
- ◯ North East and Yorkshire
- ◯ North West
- ◯ South East
- ◯ South West

Q31. What is your sex? A question about gender identity will follow.

- ◯ Female
- ◯ Male
- ◯ Prefer not to say

Q32. Is the gender you identify with the same as the sex you were registered at birth?

- ○ Yes
- ○ No ____
- ○ Prefer not to say

Q33. What is your ethnic group? Choose one option that best describes your ethnic group or background.

White

- ○ English, Welsh, Scottish, Northern Irish or British
- ○ Irish
- ○ Gypsy or Irish Traveller
- ○ Roma
- ○ Any other white background

Mixed/multiple ethnic groups

- ○ White and Black Caribbean
- ○ White and Black African
- ○ White and Asian
- ○ Any other mixed or multiple ethnic background

Asian/Asian British

- ○ Indian
- ○ Pakistani
- ○ Bangladeshi
- ○ Chinese
- ○ Any other Asian background

Black / African / Caribbean / Black British

- ○ African

○ Caribbean

○ Any other Black, African or Caribbean background

Other ethnic group

○ Arab

○ Any other ethnic group

○ **Prefer not to say**

Q34. Is there anything else about your role as Patient Safety Specialist you would like to add?

____

____

____

____

____
